# Elevated expression of serum soluble ST2 in clinical relapse after stopping long-term Nucleos(t)ide analogue therapy for chronic hepatitis B

**DOI:** 10.1186/s12879-019-4261-3

**Published:** 2019-07-19

**Authors:** Linqing Xie, Guichan Liao, Hongjie Chen, Muye Xia, Xuan Huang, Rong Fan, Jie Peng, Xiaoyong Zhang, Hongyan Liu

**Affiliations:** 1 State Key Laboratory of Organ Failure Research, Guangdong Provincial Key Laboratory of Viral Hepatitis Research, Department of Infectious Diseases, Nanfang Hospital, Southern Medical University, Guangzhou, China; 2 Hepatology Unit and Department of Infectious Diseases, Nanfang Hospital, Southern Medical University, 1838 Guangzhou Avenue North, Guangzhou, 510515 China

**Keywords:** Chronic hepatitis B, Nucleos(t)ide analogue therapy, Treatment cessation, Clinical relapse, Serum soluble ST2

## Abstract

**Background:**

The virological or clinical relapse is common in chronic hepatitis B (CHB) patients after stopping long-term nucleos(t)ide analogue (NA) therapy. Soluble growth stimulation expressed gene 2 (sST2), one of the Toll-like/interleukin-1 receptor members, is involved in a variety of inflammatory processes and immune responses. However, the expression and function of serum sST2 in CHB patients after stopping NA treatment remains unknown.

**Methods:**

A total of 91 non-cirrhotic Asian patients with CHB who discontinued NA therapy according to international guidelines were prospectively followed up to 240 weeks. All patients were divided into clinical relapse group and non-clinical relapse (including sustained virological response and only virological relapse) group according HBV DNA and ALT levels. The serum levels of sST2 of all participants were determined by ELISA and compared between each two groups.

**Results:**

Clinical relapse occurred in 26 patients and virological relapse occurred in 57 patients. We found that there was a positive correlation between sST2 expression and HBsAg, ALT, HBV DNA, and anti-HBc levels in CHB patients after discontinuation of NA treatment. Levels of serum sST2 in clinical relapse patients showed a rising trend and most patients showed peak sST2 levels at the point of clinical relapse. Moreover, the sST2 levels of clinical relapse group at week 12, week 24 and week 48 were relatively higher than non-clinical relapse group. However, the level of sST2 at the end of treatment was not an effective biological marker for the early prediction of clinical relapse after discontinuation of long-term NA therapy.

**Conclusions:**

In conclusion, we found that an increase in sST2 in clinical relapse patients might be associated with an inflammation-related immune response after discontinuation of NA treatment.

**Trial registration:**

The trial was retrospectively registered at Chinese Clinical Trial Registry: ChiCTR-OOC-17013970. Registration date: December 15, 2017.

## Background

Hepatitis B virus (HBV) infection causes acute and chronic hepatitis and remains a public health problem across the world. It is estimated that 240 million people suffers HBV infection and their prevalence is geographically different. Every year, HBV-associated end-stage liver disease results in approximately one million deaths [1]. Currently, nucleos(t)ide analogue (NA) therapy is recommended by international clinical guidelines in chronic hepatitis B (CHB) patients [2, 3]. Effective suppression of HBV DNA using long-term NA therapy has been proven to delay disease progression in patients with CHB [2]. But discontinuation of NA treatment is often associated with HBV rebound and recurrence of active hepatitis after anti-HBe seroconversion [4]. Generally, HBsAg loss which links to host immune control is considered as a satisfactory biomarker for functional cure [2, 3]. However, it has been suggested that patients who achieve hepatitis B surface antigen (HBsAg) loss could discontinue NA treatment, but HBsAg loss is rare, even after long-term NA treatment [5, 6].

Currently, many studies have demonstrated that patients have very high rates of virological or clinical relapse after NA treatment cessation [5–7]. Related studies have identified that several factors could be used for predicting relapse after treatment discontinuation, including patient age [8], serum HBsAg [9], consolidation treatment time [10], residual HBV DNA levels [11], hepatitis B virus core-related antigen (HBcrAg) levels [12], and intrahepatic HBV covalently closed circular DNA (cccDNA) levels at the end of therapy [13]. There is still lack of other reliable immune biomarkers to predict early relapse after stopping long-term NA therapy.

Growth stimulation expressed gene 2 (ST2) is a member of the Toll-like/interleukin-1 receptor superfamily [14, 15]. It has two isoforms: transmembrane ST2 expressed on the cell surface and soluble ST2 (sST2) in the serum [16]. sST2 expression is elevated in several inflammatory diseases, including atopic individuals with allergic symptoms or exacerbation of asthma [17, 18], atopic dermatitis [19], rheumatoid arthritis [20, 21], ulcerative colitis, Crohn’s disease [22], and systemic lupus erythematosus [23]. In liver diseases, sST2 was reported to positively correlates with alanine aminotransferase (ALT) levels in chronic hepatitis patients [24]. And it has been proved to be a promising prognostic biomarker in HBV-related acute-on-chronic liver failure [25].

Previous studies from our group had demonstrated that after NA treatment discontinuation, a lower clinical relapse rate was observed in younger patients and in those with low end-of treatment HBsAg levels. And the persistence of off-treatment elevated HBV DNA levels were useful in the prediction of clinical relapse and may be used to guide off-treatment management [9]. Moreover, serum levels of anti-HBc might be used to select patients suitable for NA treatment discontinuation [26]. However, the expression and role of sST2 in patients after discontinuation of long-term NA therapy has yet to be explored. This prospective study investigated the expression and clinical significance of sST2 in CHB patients after cessation of long-term NA therapy.

## Methods

### Study subjects

Ninety-one CHB patients who enrolled in a prospective, single-center, observational study and underwent cessation of NA therapy (Chinese Clinical Trial Registry number: ChiCTR-OOC-17013970) were included in this study. Patients were recruited from Nanfang Hospital (Guangzhou, China) from November 2012 until May 2018 and provided informed consent and voluntarily entered the study cohort [27]. Patients discontinued NA therapy in accordance with the 2012 The Asian Pacific Association for the Study of the Liver (APASL) guideline [28]. The criterion of discontinuation NA therapy for CHB patients was HBeAg seroconversion, undetectable HBV DNA, and normalized ALT level, and consolidated treatment for at least 12 months or 18 months. Patients who were co-infected with hepatitis C virus, hepatitis D virus, or human immunodeficiency virus were excluded.

### Follow-up

Patients enrolled in the study were followed up once a month within first 3 months. Thereafter, the patients were followed up every 3 months. After 2 years, the patients were followed up every 6 months. At each timepoint, blood samples were collected for biochemical and virological parameters examination.

### End points, retreatment, and definitions

The main clinical endpoint of this clinical trial was clinical relapse (CR), defined as: HBV DNA > 2000 IU/ml, combined with ALT >2ULN. The patients who experienced clinical relapse were withdrawn from the follow-up study and retreated using NA. The rest of the patients were defined as the non-clinical relapse (NCR) group. The non-clinical relapse group include the virological relapse group and the sustained virological response group. The virological relapse was defined as: HBV DNA > 2000 IU/ml with normal ALT levels.

### Laboratory tests

An Olympus AU5400 automatic biochemical analyzer was used for biochemical detection. The ULN of the ALT level was 40 U/L for males and 35 U/L for females. Quantitative analysis of HBV DNA was performed using the Cobas HBV-specific TaqMan polymerase chain reaction assay with a lower limit of detection (LLOD) of 20 IU/ml (Roche Diagnostics, Basel, Switzerland). Serum HBsAg (LLOD, 0.05 IU/ml) and HBeAg quantitation were performed using an Architect Assay (Abbott Laboratories, Chicago, IL). Quantitative total serum anti-HBc (IgG and IgM) was tested with a double-sandwich immunoassay (Wantai, Beijing, China; LLOD: 0.1 IU/ml) [29].

### Enzyme-linked immunosorbent assay (ELISA)

Serum samples were stored at − 20 °C after centrifugation until use. The concentration of sST2 was quantitated using a commercial human sST2 ELISA kit (R&D Systems, USA) in accordance with the manufacturer’s instruction.

### Statistical analysis

Continuous data are expressed as either the median (minimum - maximum) or the mean ± SEM. An independent samples t test, the Mann-Whitney U test or the Chi square test were used in group comparisons. To examine the dynamic change of sST2 after cessation of long-term NA therapy, the paired T test and the repeated measures ANOVA with Bonfreroni test were used to compare the baseline sST2 levels with other different timepoints, and the Mann-Whitney U test was used to compare the sST2 between the CR group and the NCR group at different timepoints. Finally, the Cox proportional hazards regression models to identify factors associated with clinical relapse after stopping long-term NA therapy. All statistical analyses were based on two-tailed hypothesis tests with a significance level of *p ≤* 0.05. All statistical analyses were conducted using SPSS 25.0 or GraphPad Prism 8.0 software.

## Results

### Demographic data and clinical characteristic

In total, 91 non-cirrhotic Asian patients with CHB who stopped NA therapy according to APSAL guidelines were prospectively followed. At the start of treatment, 61 patients were HBeAg positive, and 30 patients were HBeAg negative. All patients met the withdrawal criteria and signed a withdrawal agreement. The withdrawal time was at least 6 months. The demographic data and clinical characteristics are presented in Table 1.

**Table 1:** Demographic data and clinical characteristics

Characteristic	*n* = 91
Baseline (end of treatment)	
Age, years	35.7 ± 7.9
Sex (Male: Female)	75:16
First-line NA therapy ^a^	39 (42%)
Therapy duration, years	56.7 ± 26.2
Consolidation therapy duration, months	28 (18–46)
ALT level, × ULN	0.5 (0.4–0.7)
ALT elevation, n (%)	
< ULN	78 (86%)
ULN-2 × ULN	12 (13%)
HBV DNA level, log_10_ IU/ml	UD
HBsAg level, log_10_ IU/ml	2.9 (2.3–3.3)
< 100 IU/ml	20 (22%)
100–1000 IU/ml	31 (34%)
> 1000 IU/ml	40 (44%)
Start of treatment	
HBeAg status (Positive: Negative)	61:30
ALT elevation ×ULN	5.33 ± 4.29
HBV DNA log_10_ IU/ml	6.20 ± 1.41

Quantitative variables are expressed as mean values ± standard deviation (range) or median values (range). *ALT* Alanine aminotransferase, *HBsAg* Hepatitis B surface antigen, *HBV* Hepatitis B virus, *NA* Nucleos(t)ide analogue, *UD* Undetectable (< 20 IU/ml), *ULN* Upper limit of normal

^a^First-line: entecavir, tenofovir

### Clinical relapse and virological relapse after stopping long-term NA therapy

**Fig. 1:**
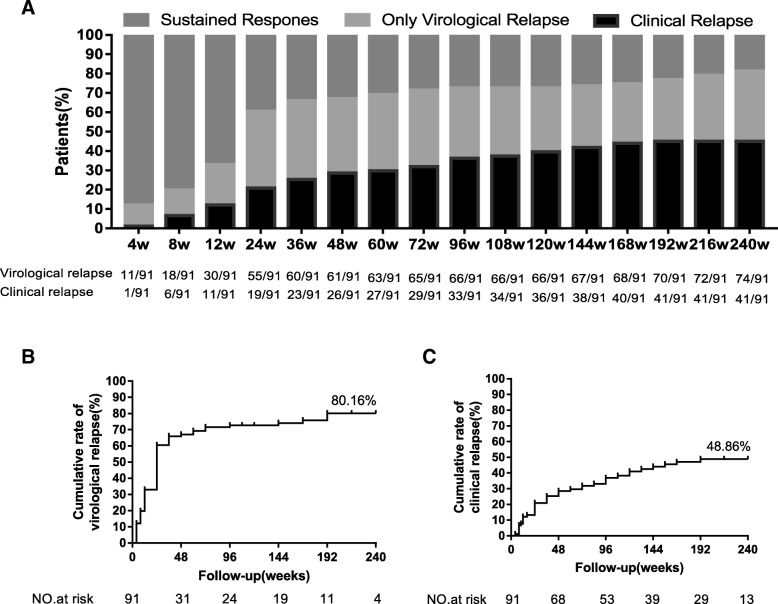
The cumulative rate of virological and clinical relapse after cessation of nucleos(t)ide analogue (NA) treatment for 240 weeks. **a** Frequency of patients in sustained response, virological relapse and clinical relapse at different timepoints. **b** The cumulative rate of virological relapse in all patients. **c** The cumulative rate of clinical relapse in all patients

All 91 patients who met the international withdrawal criteria were followed up to 240 weeks. The ratio of clinical relapse, virological relapse, and sustained response at each time point is shown in Fig. 1a. The cumulative virological relapse rate was 80.16% (Fig. 1b), and the cumulative clinical relapse rate was 48.86% (Fig. 1c), which was consistent with previous findings [5–7]. Moreover, it could be found that the majority of virological relapse (61/91, 67.03%) and clinical relapse (26/91, 28.57%) occurred within 48 weeks.

### Dynamic change of serum sST2 concentrations after cessation of long-term NA therapy

**Fig. 2:**
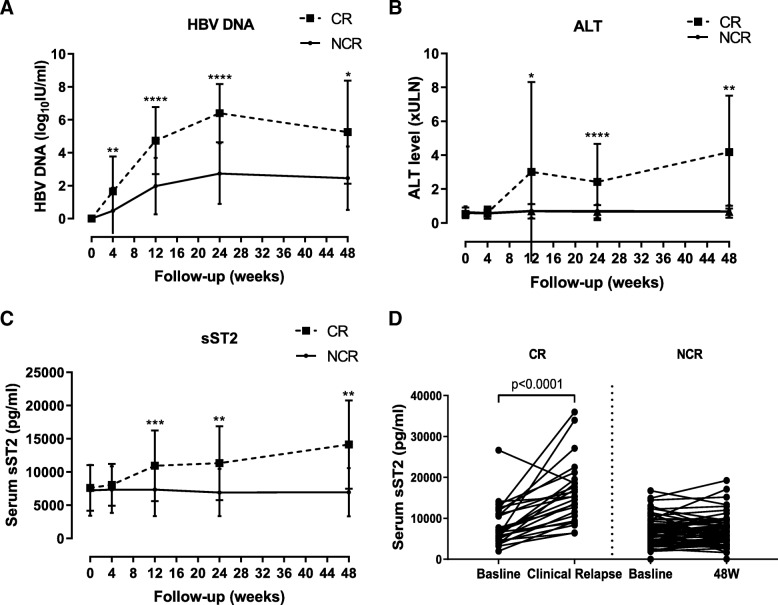
Longitudinal analysis of serum sST2 expression in 91 CHB patients after stopping long-term NA therapy for 48 weeks. **a** The dynamics of HBV DNA levels in serum in the CR and NCR group. **b** The dynamics of ALT levels in serum in the CR and NCR group. **c** The dynamics of sST2 levels in serum in the CR and NCR group. **d** Changes in serum sST2 between baseline and the point of clinical relapse in CR group and changes in serum sST2 between baseline and 48 W in NCR group. * *p* < 0.05, ** *p* < 0.01, *** *p* < 0.001, **** *p* < 0.0001

Then, we examined the concentration of serum sST2 by ELISA in patients after discontinuation of NA therapy for 48 weeks. The CHB patients were divided into CR and NCR group depending on their ALT and HBV DNA levels before week 48. Patients in the CR group were older than those in the NCR group (*p* = 0.039) and had relatively higher end-of-treatment HBsAg levels (*p* = 0.071). Pre-treatment HBeAg status, ALT, and HBV DNA levels no significant differences between CR and NCR group (Table 2). After stop of treatment, the level of the HBV DNA was increased for 24 weeks and slightly decreased during 24–48 weeks in CR group. In the NCR group, HBV DNA levels have the same trend, but is lower than the CR group at each timepoint (Fig. 2a). ALT levels were significant higher at week 24 and week 48 in CR group than NCR group (Fig. 2b). These results indicated that most patients develop clinical relapse after discontinuation of cessation of NA treatment at earlier time.

**Table 2:** Clinical characteristics of the subjects after stopping long-term NA therapy (longitudinal study)

Variable	Group	CR	NCR	χ^2^/Z	P
N		26	65		
Gender (M/F)		20/6	55/10	0.758	0.384^a^
Age (years)		39 (25–60)	35 (18–49)	−2.063	0.039^b^
	HBV DNA (log_10_ IU/ml)	UD	UD	–	–
	ALT (× ULN)	0.488 (0.30–1.00)	0.519 (0.15–1.53)	−0.868	0.385^b^
	HBsAg (log_10_ IU/ml)	2.995 (1.56–4.37)	2.810 (−2.00–4.14)	−1.805	0.071^b^
	Anti-HBc (log_10_ IU/ml)	2.471 (1.51–3.64)	2.718 (1.21–3.86)	− 1.162	0.245^b^
Week 4	HBV DNA (log_10_ IU/ml)	0.00 (0.00–5.87)	0.00 (0.00–5.92)	−3.233	0.001^b^
	ALT (× ULN)	0.543 (0.25–2.08)	0.475 (0.20–1.55)	−0.545	0.586^b^
	HBsAg (log_10_ IU/ml)	2.980 (1.47–4.37)	2.815 (−2.00–4.13)	−1.651	0.099^b^
	Anti-HBc (log_10_ IU/ml)	2.585 (1.41–3.17)	2.793 (1.21–3.76)	−1.296	0.195^b^
Week 12	HBV DNA (log_10_ IU/ml)	3.98 (2.41–8.93)	2.39 (0.00–5.71)	−5.093	< 0.001^b^
	ALT (× ULN)	0.688 (0.30–18.20)	0.615 (0.15–1.98)	−1.456	0.145^b^
	HBsAg (log_10_ IU/ml)	3.125 (2.12–.75)	2.760 (−1.70–4.14)	−2.705	0.007^b^
	Anti-HBc (log_10_ IU/ml)	3.409 (1.77–4.71)	2.972 (1.21–4.43)	−1.555	0.12^b^
Week 24	HBV DNA (log_10_ IU/ml)	5.99 (4.30–8.93)	3.110 (0.00–5.73)	−5.432	< 0.001^b^
	ALT (× ULN)	1.074 (0.55–8.40)	0.550 (0.16–2.03)	−3.929	< 0.001^b^
	HBsAg (log_10_ IU/ml)	3.575 (2.36–4.81)	2.680 (−2.00–4.19)	− 3.756	< 0.001^b^
	Anti-HBc (log_10_ IU/ml)	3.634 (2.14–4.43)	3.344 (1.30–4.95)	−2.068	0.039^b^
Week 48	HBV DNA (log_10_ IU/ml)	5.66 (0.00–7.95)	2.880 (0.00–5.87)	−2.391	0.017^b^
	ALT (× ULN)	3.200 (1.45–7.90)	0.575 (0.18–1.80)	−2.879	0.004^b^
	HBsAg (log_10_ IU/ml)	3.450 (2.77–3.45)	2.695 (−2.00–4.07)	−1.657	0.097^b^
	Anti-HBc (log_10_ IU/ml)	4.395 (4.13–4.71)	3.378 (1.43–4.97)	−3.622	< 0.001^b^
Start of treatment					
HBeAg (Positive:Negative)		17/9	46/19	0.253	0.615
ALT elevation ×ULN		3.53 (0–13.57)	6.20 (0–56.1)	−1.538	0.124
HBV DNA log_10_ IU/ml		6.17(4.07–8.76)	6.33(3.32–9.15)	−0.371	0.711

Values are n or median (range)

^a^Chi - squared test

^b^Mann -Whitney *U*-test

*M/F* Male or female, *CR* Off-treatment clinical relapse, *NCR* Non-clinical relapse group; *ALT* Alanine aminotransferase, *HBsAg* Hepatitis B surface antigen, *HBV* Hepatitis B virus, *UD* Undetectable (< 20 IU/ml), *ULN* Upper limit of normal

We further compared serum sST2 levels in the CR group and NCR group at week 0, week 4, week 12, week 24, and week 48 after discontinuation of NA therapy. There was no significant difference between the two groups at baseline and week 4, and the CR group expressed higher sST2 levels then NCR group at week 12, week 24 and week 48 (Fig. 2c). By using repeated measures ANOVA analysis, we only observed the significant difference of serum sST2 levels in the CR group between week 48 and week 12 (*p* = 0.004), week 48 and week 0 (*p* = 0.007), respectively. Further compared with the baseline at the end of treatment, sST2 expression was increased at clinical relapse timepoint in CR group (*p* <  0.001), while there was no significant difference between baseline and 48 W sST2 in the NCR group (Fig. 2d).

### Serum sST2 expression is positively correlated with HBsAg, ALT, HBV DNA and anti-HBc levels

**Fig. 3:**
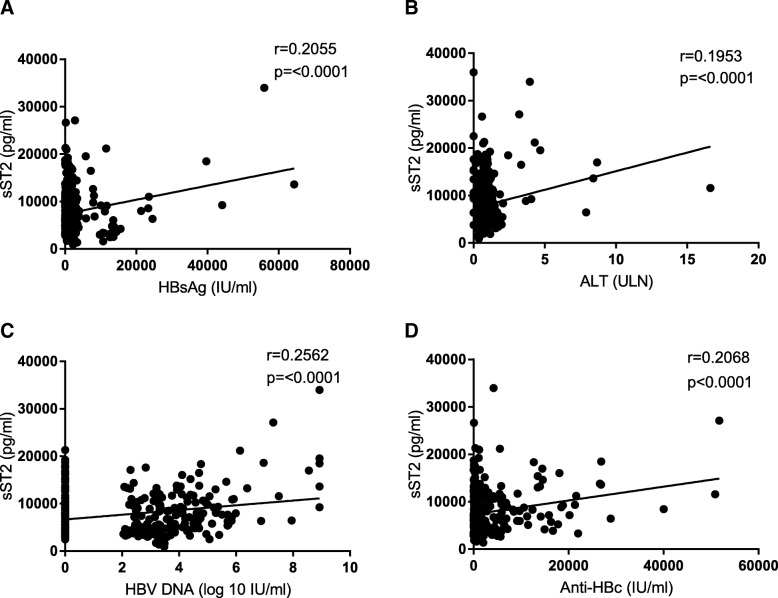
The correlation between serum soluble ST2 (sST2) and clinical parameters among all patients at every follow-up timepoint. **a** Pearson’s correlation of HBV surface antigen (HBsAg) levels and sST2 expression in serum among all patients at every follow-up timepoint. **b** Pearson’s correlation analysis of alanine aminotransferase (ALT) levels and sST2 expression in serum among all patients at every follow-up timepoint. **c** Pearson’s correlation analysis of HBV DNA levels and sST2 expression in serum among all patients at every follow-up timepoint. **d** Pearson’s correlation analysis of antibodies against hepatitis B core protein (anti-HBc) levels and sST2 expression in serum among all patients at every follow-up timepoint

Pearson analysis was used to evaluate the association between serum sST2 with HBsAg, ALT, HBV DNA and anti-HBc levels in patients after discontinuation of NA treatment. As shown in Fig. 3, there were positive correlations between serum sST2 levels and HBsAg (*r* = 0.2055, *p* <  0.0001, Fig. 3a), ALT (*r* = 0.1953, *p* <  0.0001, Fig. 3b), HBV DNA (*r* = 0.2562, *p* <  0.0001, Fig. 3c) and anti-HBc (*r* = 0.2068, *p* <  0.0001, Fig. 3d). These data further suggested that sST2 expression might be associated with liver inflammation caused by virus replication rebound during clinical relapse.

### Baseline sST2 expression could not predict clinical relapse after stopping of NA treatment

To elucidate whether serum sST2 levels at the end of treatment were associated with clinical relapse follow-up to 240 weeks, univariate and multivariate Cox regression analyses were conducted. As shown in Table 3, the age > 35 y (*p* = 0.006; hazard ratio [HR]: 2.647, 95% confidence interval [CI]: 1.324–5.291), HBsAg > 200 IU/ml (vs ≤ 200 IU/ml)(*p* = 0.006; HR: 3.697, 95% CI: 1.447–9.444),HBV DNA level elevation > 20000 IU/ml (vs ≤ 20000 IU/ml) (*p* = 0.017; HR: 2.531, 95% CI: 1.179–5.435) and the levels of sST2 at week 12 (*p* <  0.001; HR: 4.655, 95% CI: 2.345–9.243) were found to be independent predictors for clinical relapse in off-treatment CHB patients. The baseline serum sST2 expression was not statistically significant to be an independent predictor of clinical relapse. However, we could observe a HR of 1.718 in the factor of sST2, indicating that the risk of clinical relapse is 1.718 times higher in patients with elevated sST2 than in patients with non - elevated sST2.

**Table 3:** Cox proportional hazards regression analysis of clinical relapse

	Clinical Relapse (*n* = 41)					
	Univariable			Multivariable^a^		
	HR	95% CI	P	HR	95% CI	P
End of treatment						
Age	1.069	1.025–1.114	0.002	1.062	1.016–1.110	0.007
Age > 35 y	2.647	1.324–5.291	0.006			
Sex	1.44	0.662–3.133	0.358			
First-line NA therapy ^b^	1.233	0.667–2.281	0.504			
Consolidation duration	0.996	0.980–1.011	0.596			
Consolidation duration 3 > y	0.777	0.401–1.508	0.456			
ALT × ULN	0.861	0.331–2.241	0.759			
HBsAg log_10_ IU/ml	1.1725	1.177–2.529	0.005	2.086	1.286–3.385	0.003
HBsAg >200IUmL (vs ≤ 200IUmL)	3.697	1.447–9.444	0.006			
HBV DNA level elevation log_10_ IU/ml	1.000	1.000–1.000	0.030	1.000	1.000–1.000	0.045
HBV DNA level elevation > 20000 IU/ml (vs ≤ 20000 IU/ml)	2.531	1.179–5.435	0.017			
sST2 log_10_ pg/mL	2.816	0.731–10.845	0.132			
sST2 > 3.7 log_10_ pg/mL(vs ≤ 3.7 log_10_ pg/mL)	1.718	0.841–3.508	0.137			
12 W sST2 log_10_ pg/mL	4.655	2.345–9.243	< 0.001	4.403	2.169–8.937	< 0.001
Start of treatment						
HBeAg	0.726	0.384–1.375	0.326			
ALT × ULN	0.986	0.935–1.019	0.403			
HBV DNA log_10_ IU/ml	0.745	0.792–1.385	0.745			

*Abbreviations*: *ALT* Alanine aminotransferase, *CI* Confidence interval, *HBeAg* Hepatitis B virus envelope antigen, *HBsAg* Hepatitis B virus surface antigen, *HBV* Hepatitis B virus, *HR* Hazard ratio, *NA* Nucleos(t)ide analogue, *ULN* Upper limit of normal

^a^Adjusted for start-of-treatment HBeAg status and factors that were statistically different between HBeAg-positive and HBeAg-negative patients (age and consolidation therapy duration)

^b^Entecavir

## Discussion

In this prospective follow-up study of Asian patients with CHB who stopped NA therapy, we demonstrated that the cumulative rate of virological relapse was higher than the cumulative rate of clinical relapse (80.16% vs 48.86). Moreover, most patients occurred virological recurrence or clinical recurrence within 48 weeks. This observation might be due to that the NA therapy, regardless of differences in antiviral potency, only has a small effect on levels of intrahepatic cccDNA. As the template for HBV RNA transcription, residual cccDNA pool or integrated viral DNA produced viral RNA continuously and viral DNA replication was recovery in infected hepatocytes after cessation of long-term NA therapy [5, 7, 30, 31]. Therefore, a high rate of virologic rebound was observed in HBsAg positive patients after discontinuation of NA treatment. To avoid rebound of HBV DNA after stopping NA therapy, the best stopping criterion might be HBsAg seroclearance, which referred to host immune control of infection [32].

It had been suggested that a relapse, resulting in increased HBV replication, could change the cytokine milieu and lead to increased responsiveness of HBV-specific T cells and NK cells at 12 weeks after stopping long-term NA treatment [33, 34]. Thus, the inflammation-associated cytokines, which correlated with the activation of host immune system, were elevated at 12 weeks after discontinuation of NA treatment [35]. In murine inflammatory models, the expression of ST2 is induced by proinflammatory stimuli [36, 37]. We found that serum sST2 was positively correlated with HBsAg, HBV DNA, ALT and anti-HBc levels. Therefore, an early virological rebound might be accompanied with a transient increase in ALT and an elevation of sST2 expression after discontinuation of the NA therapy. In addition, serum sST2 levels in the CR group were found to be higher than in the NCR group after discontinuation of NA treatment. Moreover, the level of sST2 at the point of clinical relapse was significantly higher than the sST2 levels at baseline. These results were consistent with previous findings that serum ST2 levels were significant higher in CHB patients than in healthy controls [38], and ST2 levels are positively correlated with ALT in immune active CHB patients [24].

Identifying the useful biomarker to predict clinical relapse after stopping long-term NA therapy remained to be a challenge for management of CHB patients. By Cox proportional hazards regression analysis, we confirmed that age and baseline HBsAg levels were factors that affecting clinical relapse, which was consistent with previous findings [39]. However, baseline sST2 expression was not an independent predictor of clinical relapse. Interestingly, the levels of sST2 at week 12 can predict the clinical relapse after stopping NA therapy. It has been shown that the host immunity, including innate and adaptive immune responses, played an important role in the control and resolution of chronic HBV infections [40]. Thus, whether HBV specific immune biomarkers could be used for prediction of clinical relapse needed further investigation.

There were some limitations to the current study. Firstly, the number of cases included in the current study is relatively small, with a total of 91 cases, and there is a need to further expand the sample size in future research. Secondly, the source of elevated sST2 expression and the function of sST2 in chronic HBV infection were not studied in this study by in vitro cell culture system. Finally, serum sST2 levels showed an upward trend in the CR group and were higher at the point of clinical relapse than at baseline, but not in the NCR group. The specific mechanism underlying this phenomenon remained to be clarified in the future.

## Conclusions

The incidence of virological relapse and clinical relapse was high in CHB patients after stopped NA therapy. The increase of sST2 in clinical relapse patients might be associated with an inflammation-related immune response after discontinuation of NA treatment.

## Data Availability

The datasets used and/or analyzed during the current study available from the corresponding author on reasonable request.

## Abbreviations

ALT: Alanine aminotransferase
anti-HBc: Hepatitis B core antibody
cccDNA: covalently closed circular DNA
CHB: Chronic hepatitis B
HBsAg: Hepatitis B virus surface antigen
HBV: Hepatitis B virus
HCC: Hepatocellular carcinoma
LLOD: Lower limit of detection
NA: Nucleos(t)ide analogue
sST2: Soluble growth stimulation expressed gene 2
ULN: Upper limit of normal
